# Intercontinental comparison of women with breast cancer treated by oncologists in Europe, Asia, and Latin America: a retrospective study of 99,571 patients

**DOI:** 10.1007/s00432-023-04681-7

**Published:** 2023-03-15

**Authors:** Arturas Kadys, Niklas Gremke, Laura Schnetter, Karel Kostev, Matthias Kalder

**Affiliations:** 1 Department of Gynecology and Obstetrics, University Hospital Marburg, Philipps-University Marburg, Marburg, Germany; 2 Oncology, IQVIA, Frankfurt Am Main, Germany; 3 Epidemiology, IQVIA, Unterschweinstiege 2–14, Frankfurt am Main, 60549 Frankfurt Am Main, Germany

**Keywords:** Breast cancer, Oncology, Intercontinental comparison, Metastatic disease, Retrospective study

## Abstract

**Purpose:**

The aim of the study was to evaluate the baseline data of women with breast cancer (BC) undergoing treatment in an intercontinental comparison.

**Methods:**

This study included 99,571 women with BC from Europe (70,834), Asia (18,208), and Latin America (10,529) enrolled between 2017 and 2021, based on data from IQVIA’s Oncology Dynamics database. This source is supplied with information by means of a cross-sectional partially retrospective survey collecting anonymized data on inpatients and outpatients treated by a representative panel of oncologists. A multivariable logistic regression model was used to investigate the probability of metastases.

**Results:**

The data available in Asia (98%) and Latin America (100%) were hospital data, while in Europe, patients were treated both in hospitals and in office-based practices (62%, 38%). The mean age in Asia and Latin America (57 ± 13) was lower than in Europe (61 ± 13; *p* < 0.001). Lobular BC was diagnosed twice as often in Europe compared to Asia and Latin America (15.2%, 9.8%, 8.0%). The number of patients with metastasized hormone receptor-positive (HR +) BC was significantly higher in Europe and Latin America than in Asia (76%, 68%; *p* < 0.001). The highest number of women with metastasized BC was reported in Europe (26% compared to 14% and 20%, respectively, in Asia and Latin America). Across the continents, the percentage of women with BC who experienced metastases was 51–61% for bone, 30–39% for lung and 25–32% for liver, followed by 3–6% for skin and 3% for brain.

**Conclusion:**

Women with BC treated in Europe tend to be significantly older and more likely to develop metastases than women in Asia and Latin America, except for lung metastases.

## Introduction

Female breast cancer (BC) is the most frequently diagnosed cancer and the leading cause of cancer mortality in women worldwide (Global Cancer Statistics 2020). In 2020, over 2.3 million patients were newly diagnosed with BC, and 685,000 death from breast cancer occurred in 2020 (Arnold et al. 2022).

Globally every fourth woman is diagnosed with BC, with every sixth woman suffering a lethal outcome (Global Cancer Statistics 2020). Developed countries such as Western and Northern Europe, North America, and Australia have the highest incidence rates for BC at more than 80 per 100,000, while South and Central Asia, Central America, and Africa have the lowest incidence rates at less than 40 per 100,000. However, incidence rates of BC are increasing rapidly in transitional countries in Latin America, Africa (Joko-Fru et al. 2020 Oct 15), and Asia (Bray et al. 2004), as well as in transitional Asian countries such as Japan and South Korea, where the number of women with BC has historically been low (Heer et al. 2020).

A rising incidence rate of hormone receptor-positive (HR +) BC has been reported based on analyses of the biological profile of the disease. A number of studies from Europe (Anderson et al. 2013; Mullooly et al. 2017; Mesa-Eguiagaray et al. 2020) and the United States (Glass et al. 2007; Anderson et al. 2011) support recent findings, indicating a notably stronger association between obesity and HR + BC (Reeves et al. 2007; Renehan et al. 2008) and addressing the potential impact of mammography screenings, which, for example, detect small, slow-growing HR + tumors (Gilliland et al. 2000).

Survival rates are directly related to the stage of BC and decrease significantly in the case of both locally advanced stage and metastatic disease. In descending order of frequency, the most common sites of distant metastasis in BC are the bones, lungs, liver, brain, and skin. The 5-year survival rate is up to 99% for locally confined disease, drops to 86% for locally advanced disease, and falls dramatically when remote metastases develop. (Noone et al. 2018).

Most studies on BC rely on national or international data and are limited to one continent at most. To the best of our knowledge, there is a lack of global databases capable of offering a statistical representation of real-world BC data. The aim of the present study was to evaluate the baseline data of women with BC undergoing treatment from an epidemiological perspective as part of an intercontinental comparison.

## Methods

### Database

This retrospective cross-sectional study is based on data from IQVIA’s Oncology Dynamics (OD) database (Alymova et al. 2022). This source is a retrospective survey of the representative panel of physicians, mainly oncologists. OD collects fully anonymized patient-level data on drug-treated cancer patients in several countries worldwide. Data collection and reporting is conducted through a standardized online questionnaire in which all questions are mandatory. Specific instructions are displayed by a ‘pop-up’ system throughout the survey to provide clear definitions for the desired variables. Physicians are also asked to enter factual information from the patient’s medical records to avoid recall biases. Further tactics to ensure input accuracy include controlled code lists and multiple-choice questions, as well as interactive filters that limit non-applicable questions (e.g., items on cancer-specific biomarkers). Responses are immediately validated against previous answers and reference files; “unexpected value” messages are displayed to participants where relevant, prompting them to double-check their response. Physicians are instructed to report the most recent consecutive cases (up to 20 cases depending on the specialty) they have treated during the last 7-day period to discourage selective case submission. No duplicate patient cases can be delivered. After form submission, additional validations and trend checks are performed; anomalous values are discussed with the participant who submitted them and corrected as needed.

### Patient selection and study outcome

Surveys of all female patients with breast cancer (ICD-10: C50) submitted between January 1, 2017 and March 31, 2021, were available for five European countries (Germany, France, United Kingdom (UK), Spain, Italy), three Asian countries (China, Korea, and Japan) and two Latin American countries (Brazil and Mexico). The outcome of the study was the proportion of breast cancer patients with a documentation of different metastases (bones, liver, lung, skin, and brain) depending on the region. Cancer stage was defined using the TNM classification system.

### Statistical analysis

Baseline characteristics were compared for subjects in different regions using Chi^2^ tests for categorical variables (age group, HR status, histology) and Wilcoxon tests for age. The prevalence of defined metastases was calculated as the proportion of patients with defined metastases in relation to all patients with stage IV breast cancer upon diagnosis and was differentiated by region. The prevalence of defined metastases was also calculated separately for patients with positive and negative HR status. To investigate the probability of metastasis, a multivariable logistic regression model was fitted with each metastasis (yes/no) as a dependent variable and region as an impact variable, adjusting for age, HR status, and histology. The results of the regression analyses are presented as odds ratios (ORs) with 95% confidence intervals (CIs). P values lower than 0.05 were considered statistically significant. All analyses were performed using SAS 9.4 (SAS Institute, Cary, US).

The OD database has been used in some retrospective studies [for example, 17–19].

## Results

This study comprised 99,571 women with BC across 3 continents: Europe 70,834 (71%), Asia 18,208 (18%), and Latin America 10,529 (11%). In Asia (98.1%) and Latin America (100%), the data available are mainly clinical, while in Europe, patients are treated in both clinics and office-based practices (61.5%, 38.5%). The mean age in Asia and Latin America (57 ± 13) is significantly lower than in Europe (61 ± 13; *p* < 0.001). Lobular breast cancer is diagnosed almost twice as often in Europe as in Asia and Latin America (15%, 9%, 8%; *p* < 0.001; Table 1). Further baseline data are displayed in Table 1.

**Table 1:** Baseline characteristics of study patients

Variable	Europe	Asia	Latin America	*P* value
N	70,834	18,208	10,529	
Hospital	61.5	98.1	100.0	< 0.001
Office-based practice	38.5	1.9	0.0	
Age (mean, SD)	61.3 (13.0)	57.1 (13.0)	56.5 (12.5)	< 0.001
Age group				
≤ 50	22.1	32.9	33.4	< 0.001
51–60	24.6	28.1	27.8	
61–70	27.6	23.1	25.0	
> 70	25.7	16.0	13.8	
HR-positive	77.5	74.3	76.3	< 0.001
Histological tumor type				
Ductal carcinoma	77.0	77.3	75.8	0.012
Lobular breast cancer	15.2	9.8	8.0	< 0.001
Other form	1.1	1.8	1.7	< 0.001
Form not defined	6.8	11.1	14.5	< 0.001

**Fig. 1:**
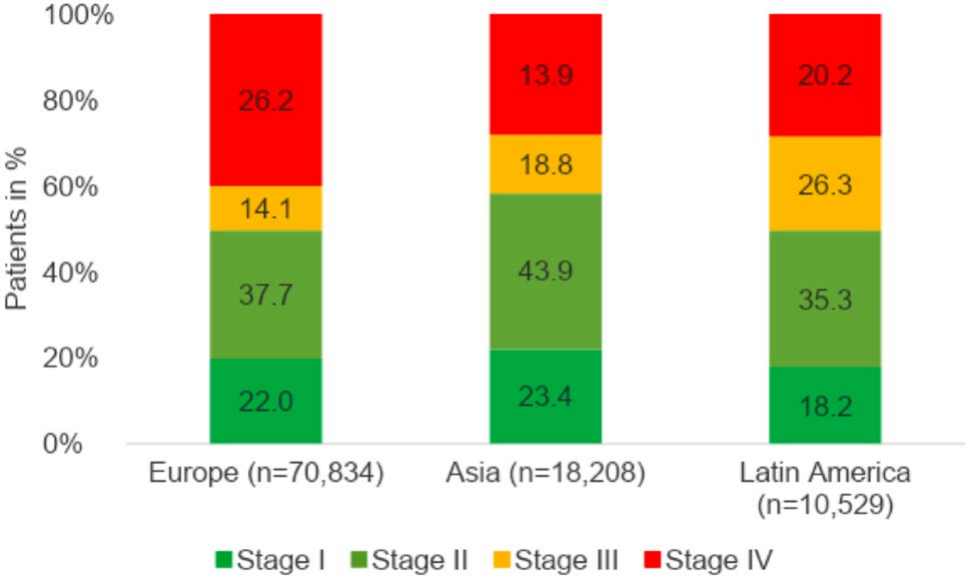
Cancer stages of study patients by continent

Figure 1 shows the intercontinental distribution of BC stages I–IV. Early-stage BC (Stages I–II) is most common in Asia (23.4%, 43.9%), followed by Europe (22.0%, 37.7%) and Latin America (18.2%, 35.3%). In Latin America, the number of patients with advanced BC (Stage III) is 26.3%, almost double that in Europe (14.1%) and Asia (18.8%). In Europe, significantly more women are diagnosed with metastasized BC (Stage IV) compared to Asia and Latin America (26.2%, 13.9%, 20.2%).

Data from this study were also compared with international epidemiological studies for lack of comparative collectives (Table 2).

**Table 2:** Global comparison of breast cancer stages, population-based data

Continent/country	Stage I	Stage II	Stage III	Stage IV
Europe				
Europe, 2010–2014 (OECD) (*n* = 2,700,348) (OECD. Health at a Glance 2020)	51	29**		10
Sweden, 2004–2009 (*n* = 3760) (Abdoli et al. 2017)	47	44	5	4
England, 2012 (*n* = 42.071) (McPhail et al.^2015^)	37	34	9	6
Europe, OD (*n* = 70,832)*	22	38	14	26
Asia				
Korea, 2018 (*n* = 10,496) (Kang et al.^2018^)	43	27	7	1
Japan, 2006 (*n* = 20,412) (Sonoo et al. 2008)	44	45	9	3
Hong Kong, 1997–2001 (*n* = 7449) (Kwong et al. 2011)	26	56	13	5
China, 1999–2008 (*n* = 4211) (Li et al. 2011 Aug)	16	45	19	2
Philippines, 1993–2002 (*n* = 7152) (Laudico et al.^2009^)	7	52	26	16
Asia, OD (*n* = 18,208) *	23	44	19	14
Latin America				
Brazil, 2000–2012 (*n* = 170,757) (Renna Junior and Silva 2018 Mar)	19	41	30	9
Mexico, 2005–2014 (*n* = 4411) ( Maffuz-Aziz et al.^2017^)	36	45**		8
Latin America, OD (*n* = 10,529)*	18	35	26	20
World				
United States, 2007–2013 (SEER 18 registries) (Surveillance, Epidemiology, and End Results^ 2016^)	49	34	11	6
United States, 2017 (*n* = 10,066) (Zimmer et al. 2018)	48	36	10	3
New Zealand, 2000–2013 (*n* = 12,390) (Seneviratne et al. 2016)	43	37	15	5

Percentages corrected to exclude stage 0 and unknown stage as well as unclassifiable BC. Percentages may not sum to 100 due to rounding

*Data from the OD database

**Data is represented as locally advanced Stage II and III BC

**Fig. 2:**
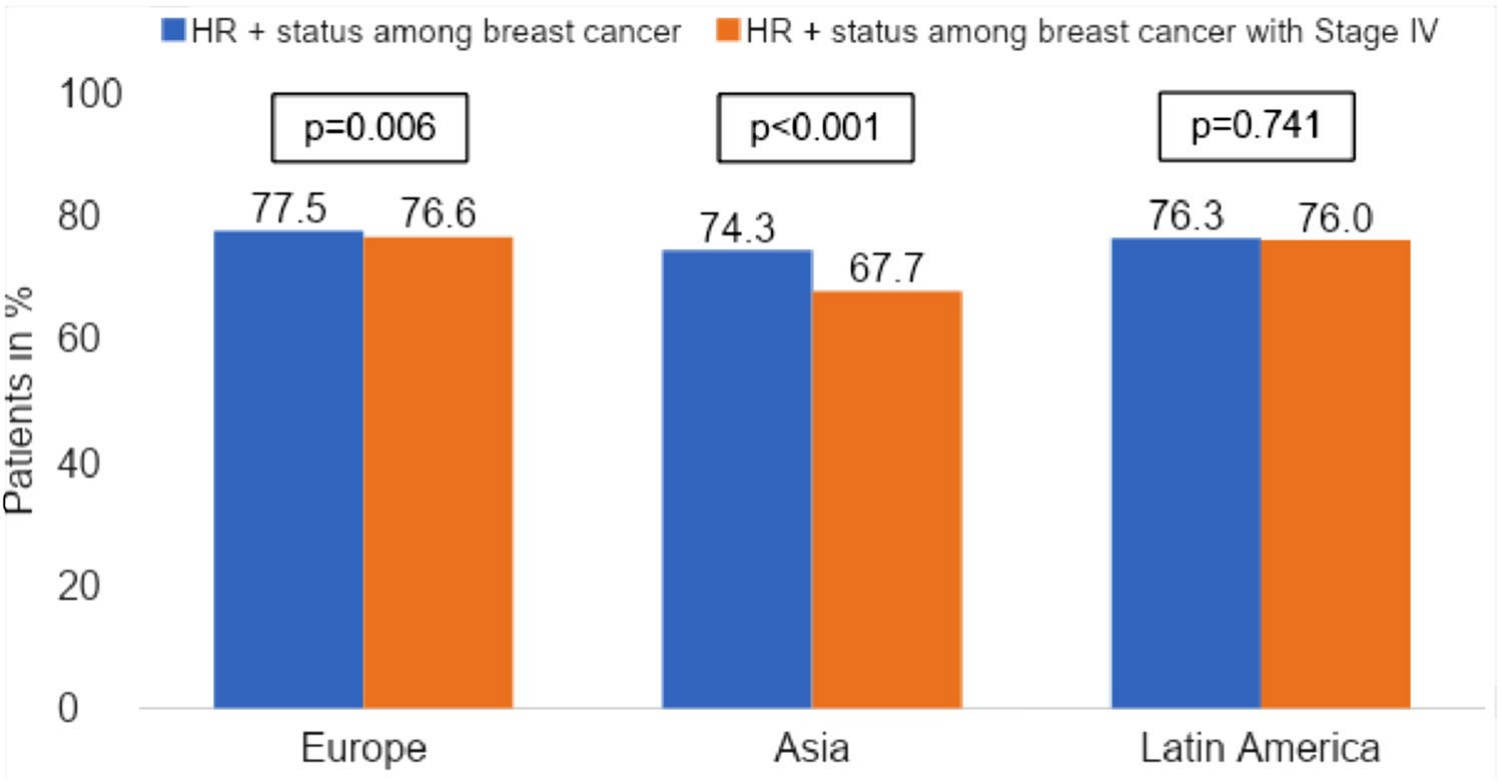
Proportion of patients with HR + status among all breast cancer patients and among Stage IV breast cancer patients

In addition, we examined the distribution of hormone receptor status in women with metastatic BC (Stage IV). In Asia, the incidence of metastatic HR-positive BC is up to 10% lower than on the other continents (see Fig. 2).

Furthermore, Table 3 shows the proportion of patients with metastases among all stage IV BC patients based on HR status. Globally, the highest prevalence of localization of metastases is observed in bone (> 50%), followed by lung (> 30%), liver (~ 30%), skin (> 4%), and brain (~ 4%).

**Table 3:** Proportion of breast cancer patients with Stage IV among all breast cancer patients differentiated by HR status

Region	Proportion of patients with metastases among all breast cancer patients (%)	Proportion of Stage IV breast cancer patients with positive HR status with metastases among all breast cancer patients (%)	Proportion of Stage IV breast cancer patients with negative HR status with metastases among all breast cancer patients (%)	*P* value
Bone				
Europe	60.8	66.7	41.5	< 0.001
Asia	50.5	54.4	42.3	< 0.001
Latin America	61.3	66.4	45.0	< 0.001
Lung				
Europe	29.8	26.6	40.0	< 0.001
Asia	38.7	38.8	38.5	< 0.001
Latin America	35.1	31.4	47.0	< 0.001
Skin				
Europe	5.8	6.0	5.0	0.015
Asia	3.6	3.3	4.4	0.155
Latin America	5.6	5.2	6.9	0.155
Liver				
Europe	29.6	25.2	44.0	< 0.001
Asia	32.2	28.9	39.2	< 0.001
Latin America	30.7	27.0	42.3	< 0.001
Brain				
Europe	3.2	2.5	5.5	< 0.001
Asia	4.3	3.8	5.5	0.049
Latin America	3.8	2.1	9.2	< 0.001

Bone metastases are reported more frequently, especially in patients with HR-positive BC, and range from 54.4 to 66.7% in an intercontinental comparison, whereas lung, liver, and brain metastases are significantly prevalent in patients with HR-negative BC (*p* < 0.001). In Asia, the distribution of BC patients with lung metastases is balanced between HR + and HR- at 38%, while in Europe and Latin America, HR − BC patients are more likely to have lung metastases than HR + BC patients (40%, 26.6%, 47%, 31.4%).

Another peculiarity occurs in Latin America in BC patients with brain metastases, which are significantly more common in HR- BC patients at 9.2% than in HR + BC patients at 2.1% (*p* < 0.001).

Finally, the association between continental region and the prevalence of metastases in BC patients overall, HR + patients, and HR- patients after calculating a multivariable logistic regression model is presented in Table 4. Europe was defined as the reference country for the statistical analysis. Significantly lower rates of bone metastasis, skin metastasis, and, in HR- BC patients, liver metastasis were found in Asia compared to Europe and Latin America (*p* < 0.006). In contrast, the rate of lung metastases was equally significantly higher in Asia and Latin America than in Europe (*p* < 0.001). There are no significant intercontinental differences for either liver or brain metastases.

**Table 4:** Association between region and the prevalence of metastases in breast cancer patients (multivariable logistic regression model)

Region	Stage IV breast cancer patients		Stage IV breast cancer patients with positive HR status		Stage IV breast cancer patients with negative HR status	
	OR (95% CI)	*P* value	OR (95% CI)	*P* value	OR (95% CI)	*P* value
Bone						
Europe	Reference		Reference		Reference	
Asia	0.71 (0.65–0.78)	< 0.001	0.56 (0.38–0.82)	0.003	0.60 (0.32–1.12)	0.109
Latin America	1.08 (0.98–1.19)	0.140	0.99 (0.73–1.35)	0.945	0.44 (0.16–1.21)	0.113
Lung						
Europe	Reference		Reference		Reference	
Asia	1.45 (1.33–1.59)	< 0.001	1.76 (1.59–1.96)	< 0.001	0.99 (0.84–1.15)	0.848
Latin America	1.32 (1.19–1.45)	0.001	1.27 (1.13–1.42)	< 0001	1.46 (1.21–1.76)	< 0.001
Skin						
Europe	Reference		Reference		Reference	
Asia	0.73 (0.58–0.91)	0.005	0.65 (0.49–0.85)	0.003	0.94 (0.66–1.36)	0.754
Latin America	1.17 (0.95–1.43)	0.137	1.04 (0.82–1.32)	0.727	1.60 (1.10–2.34)	0.015
Liver						
Europe	Reference		Reference		Reference	
Asia	0.94 (0.86–1.03)	0.193	1.02 (0.91–1.14)	0.751	0.81 (0.69–0.94)	0.006
Latin America	0.96 (0.86–1.06)	0.371	0.97 (0.86–1.10)	0.662	0.91 (0.76–1.10)	0.342
Brain						
Europe	Reference		Reference		Reference	
Asia	1.04 (0.84–1.29)	0.729	1.18 (0.90–1.56)	0.229	0.89 (0.64–1.24)	0.489
Latin America	0.93 (0.73–1.19)	0.552	0.61 (0.42–0.88)	0.008	1.49 (1.07–2.09)	0.020

## Discussion

A total of 99,571 women were included in this first retrospective study on three continents (Europe, Asia, and Latin America), representing 1% of the global female population with primary diagnoses of BC under treatment. The main findings were that women with BC treated in Europe tend to be significantly older than women in Asia and Latin America and more likely to develop metastases, except for lung metastases. Lobular breast cancer is diagnosed almost twice as often in Europe as in Asia and Latin America. Finally, after applying a multivariable logistic regression model, significantly lower rates of bone metastasis, skin metastasis, and, in HR- BC patients, liver metastasis were found in Asia compared to Europe and Latin America.

To the best of our knowledge, there is a lack of data from both international and intercontinental comparisons of women with BC under treatment. This study draws on unique intercontinental records of therapy from collective databases consisting of inpatient and outpatient data with a focus on clinical cases in Latin America and Asia, while European records include considerably more outpatient cases.

The age distribution of BC patients in Europe was found to be similar across all age groups, with a peak in the 60–69 years age group, and was comparable to that in the United States (DeSantis et al. 2019) and other Western countries ( McPhail et al. 2015; Seneviratne et al. 2016). In contrast, a higher number of BC cases occurred in younger patients in both Asia and Latin America (< 50 years) and there is an obvious age-associated reduction of incidence in the elderly population (Mizukoshi et al. 2020). Whether this is due to better diagnostics or early detection compliance or earlier occurrence of disease in younger women remains unclear.

In this study, patients with HR-positive BC account for more than three-quarters of the BC population and predominate in stage IV on all continents studied. Findings from studies in Denmark (Anderson et al. 2013), Ireland (Mullooly et al. 2017), Scotland (Mesa-Eguiagaray et al. 2020), and the United States (Glass et al. 2007; Anderson et al. 2011) using cancer registry data supplemented with additional information on tumor markers have shown similar results. Increasing incidence is confined to HR-positive cancer, while the rates of HR-negative cancers are falling. ER-positive breast cancer was also the most common subtype among women in Japan (76%), Korea (67%), and Hong Kong (66%) and among Israeli Jews (59%) and Malaysian and Singaporean Chinese women (57%). This may explain the finding in the present study that the proportion of HR-positive BC patients is up to 10% lower in Asia than on other continents (Kim et al. 2015). Increasing rates of HR-positive BC seem to be related to increases in obesity in the population (Allemani et al. 2013), as well as the growing impact of mammographic screening, which preferentially detects slow-growing ER-positive cancers (Gilliland et al. 2000).

Ductal carcinoma has the highest prevalence of the tumor types included in this study, accounting for up to 77% of the population, followed by lobular type BC, which varies from 8 to 15% intercontinentally. A similar distribution is found in other studies, in which ductal carcinoma reaches 79–84% and lobular BC varies by 8–12%. (OECD. Health at a Glance 2020; Li et al. 2011; Renna Junior and Silva 2018).

In the absence of comparable collectives, the results of this study were compared with other large national and international studies with an epidemiological background. As is to be expected, the epidemiological studies have a higher incidence of localized BC, because they often use data with results of mammography screening, unlike the present study (McPhail et al. 2015; Abdoli et al. 2017). As anticipated, more patients in stages of advanced BC are treated in oncology practices and centers as a result of required maintenance therapy. This may also explain the differences between this study and other epidemiological studies on metastatic BC. However, we did observe similar results to those of international studies in patients with locally advanced BC.

In high-income countries in Asia such as Korea and Japan, the vast majority of patients with BC are diagnosed at stage I or II, which is consistent with findings in Europe and the United States (McPhail et al. 2015; Kim et al. 2015; Allemani et al. 2013). In low-income Asian countries and transitioning countries, the number of patients with locally advanced and metastatic BC is significantly higher (Kim et al. 2015; Li et al. 2011; Kang et al.^2018^).

Although there are currently younger patients and a greater number of women with locally advanced breast cancer in Latin America, the trend toward predisposing changes in early detection is also noteworthy. The results of this study are comparable with those of a Brazilian study by Renna Junior NL et al. with clinically based data showing at least a twofold lower incidence of patients with early BC, and therefore, a greater number of patients with locally advanced BC compared to Western countries (Renna Junior and Silva 2018). In a study performed in Mexico, Maffuz-Aziz A et al. reported similar results, but the difference is not obvious due to the different classification used, where 23% of Stage IIA patients were combined with Stage I patients and considered early stage ( Maffuz-Aziz et al.^2017^). Compared to the study by Maffuz-Aziz A et al., the present study showed considerably higher levels of stage IV BC in Latin America. This shift to more advanced tumor stages suggests that the data source for this study is looking at patients receiving treatment, which is generally required for life in those with the metastatic disease.

Consistent with the literature, bone metastases in women with BC are found equally in more than half of the studied population on all continents (Kennecke et al. 2010; Wu et al. 2016). For instance, Wang et al. studied 18,322 women with stage IV BC between 2010 and 2015 as part of the United States SEER registry. The largest subgroup was bone metastases, comprising some 39.8% (7292) of all patients, followed by multiple metastases (33.07%, 6059), lung metastases (10.94%, 2005), liver metastases (7.34%, 1346), other metastases (7.34%, 1344), and brain metastases (1.51%, 276) (Wang et al. 2019). Unlike the present database, the SEER registry does not consider patients with multiple BC metastases as a distinct group, which could explain the lower prevalence of this subgroup.

In line with the results of several previous studies, both bone and lung metastases are common in women with HR-positive BC, while liver and brain metastases are most prevalent in women with HR-negative BC (Wang et al. 2019; Liede et al. 2016). Martin et al. reported the highest incidence of brain metastases in patients with HR − /HER2 + and triple-negative subtypes (11.37% and 11.45%, respectively). Overall, brain metastases were reported in 7.56% of women with metastatic disease and in 0.41% of the entire cohort (Martin et al. 2017). In the present study, a lower percentage of brain metastases was found in women with BC, ranging from 3.2% in Europe to 3.8% in Latin America and 4.3% in Asia. Although women with HR- BC also did not reach the high percentage indicated in the SEER database, in Latin America the percentage was twice as high as in Europe and Asia (9.2%, 5.5%, respectively). The reason for these differences may be that the present study used real-time data reported during patient treatment, rather than data from a retrospective registry database.

### Strengths and limitations

The major strength of the present study is the uniqueness of the database, with a considerable number of patients from several continents, which makes it possible to investigate and compare intercontinental differences in patients with BC on therapy for the first time. However, our study is subject to a number of limitations that should be noted. The original questionnaire was not designed for research purposes. Therefore, variables such as genetic aspects, risk factors, and socioeconomic status are missing. It is also important to note that only drug-treated patients are included in OD database, which means that the proportion of Stage III and IV patients may be higher in the database than in the general population of breast cancer patients. Further limitation is the comparison of hospital based only versus hospital and office-based samples what can cause biases. Finally, there is a further potential selection bias due to non-population-based recruitment via selective care providers.

## Conclusions

To the best of our knowledge, there is a lack of data involving an intercontinental comparison from an epidemiological perspective of women with BC undergoing treatment either in a clinical setting or in private practice. Our study showed that women with BC being treated in Europe tend to be significantly older and more likely to have metastases than women in Asia and Latin America, except for lung metastases. We also discussed the data in relation to data drawn from national registries and international epidemiological studies. Further research and database development on a global scale with a focus on cancer patients should be considered.

## Data Availability

Anonymized raw data are available upon reasonable request.
